# Research on Splitting-Tensile Properties and Failure Mechanism of Steel-Fiber-Reinforced Concrete Based on DIC and AE Techniques

**DOI:** 10.3390/ma15207150

**Published:** 2022-10-14

**Authors:** Tao Luo, Xiaofeng Pan, Liyun Tang, Qiang Sun, Jiaojiao Pan

**Affiliations:** 1 School of Civil Engineering, Xijing University, Xi’an 710123, China; 2 Shaanxi Key Laboratory of Safety and Durability of Concrete Structures, Xi’an 710123, China; 3 Architecture and Civil Engineering School, Xi’an University of Science and Technology, Xi’an 710054, China; 4 College of Geology and Environment, Xi’an University of Science and Technology, Xi’an 710054, China; 5 Shaanxi Provincial Key Laboratory of Geological Support for Coal Green Exploitation, Xi’an 710054, China

**Keywords:** steel-fiber-reinforced concrete, splitting-tensile strength, acoustic emission, digital image correlation, failure mechanism

## Abstract

Concrete presents different internal micro-structure and damage characteristics because of the different content of steel fibers and the randomness of its distribution. Therefore, the failure process of steel-fiber-reinforced concrete (SFRC) should be divided into different stages and the damage types should be classified to further clarify the strengthening mechanism of steel fibers. The role of volume fractions of steel fibers in the splitting-tensile strength of concrete was investigated by split tensile tests for concrete with four different volume fractions of steel fibers (0.0%, 1.0%, 1.5%, 2.0%). The acoustic emission energy and horizontal displacement of concrete in the splitting-tensile process were monitored by combing digital image correlation (DIC) and acoustic emission (AE) techniques, and the microscopic failure mechanism of SFRC was analyzed emphatically. The results showed that the addition of steel fibers improved the splitting-tensile strength of concrete. With the increase of the volume fraction of steel fibers, the splitting-tensile strength of concrete increased first and then decreased, and reached the maximum value of 5.294 MPa when the content was 1.5%. It was observed that the overall failure mechanism could be divided into four stages: slow accumulation of elastic energy (I); rapid accumulation of elastic energy (II); rapid accumulation of dissipated energy (III); a slow decrease of elastic energy and a slow increase of dissipated energy (IV). Tensile failure dominated the failure process of concrete splitting-tensile resistance, while there was a part of shear failure.

## 1. Introduction

In recent years, SFRC has been widely used in some fields including highway bridges, hydraulic dams, ports, and marine engineering. Due to the significant brittleness and low tensile strength of ordinary concrete, adding an appropriate amount of steel fibers can effectively improve its tensile and flexural strength [1,2], crack resistance [3], impact resistance [4,5], and fatigue resistance [6,7,8]. As the basic mechanical index of concrete, the splitting-tensile strength is the main basis for determining the cracking resistance of concrete in a structural design [9]. Scholars at home and abroad have studied the improvement of steel fibers on the tensile properties of concrete and explored the effect of parameters such as steel fiber type and length-to-diameter ratio on the splitting and tensile properties of concrete through experimental studies and theoretical calculations [10,11,12,13,14]. Ding et al. found that the splitting-tensile strength of self-compacting SFRC is determined by the steel fiber factor, and proposed the prediction model of considering the influences of steel fiber distribution and volume fractions [15]. The tensile softening law (tension functions) that can describe the post-cracking behavior of concrete made with different steel fiber volume fractions was established and the effect of steel fiber volume fractions on the shear capacity and strength gain was investigated by Kachouh et al. [16]. Material properties of structural lightweight waste aggregate concrete with different amounts of fibers (0.0%, 1.0%, and 1.5%) were presented by Lehner et al. The results showed that a greater amount of fibers reduces the service life as well as the preloading of the structure [17]. However, the characterization of the crack development and the crack-arresting mechanism of steel fibers based on theoretical methods and traditional tests is complex and economically inefficient.

The DIC technique has been widely used in material strain measurement and crack propagation because of its simple operation, lack of contact, and high precision [18]. Li et al. [19] conducted a three-point bending test on concrete, obtained the strain field and displacement field of the surface by DIC, and summarized the morphology and crack opening displacement of the fracture process zone (FPZ) at different stages. The DIC technique is able to record the crack propagation process, but it cannot characterize the energy dissipation pattern in this process [20]. The AE technology is able to quickly and accurately detect micro-damage inside the material before macroscopic damage occurs on the surface of the material by the AE signal and is based on the stress wave propagating through the solid material when the material is subjected to strain [21]. Therefore, the combination of DIC and AE is a suitable method to monitor the energy dissipation and crack propagation during the entire damage and failure process of quasi-brittle materials such as concrete [22]. Rouchina et al. [23] used both DIC and AE to monitor the loading test of fibrous mortar specimens, and the results showed that the two techniques achieved good correlation in the progressive damage development of fibrous mortar. Liu et al. [24] obtained the displacement field on the surface of the specimen and the AE signal inside the specimen based on DIC and AE techniques, which indicated that the internal and surface characteristics of FPZ evolution were consistent during the propagation of concrete cracks. Alam et al. [25] applied these two techniques to quantitatively study the propagation of the FPZ, and the results showed that the FPZ length calculated by the crack opening (DIC) was larger than that calculated by the length of energy dissipation zone (AE). Skarżyński et al. [26] studied the fracture behavior of reinforced concrete beams under quasi-static, three-point bending through these two techniques, and analyzed the influence of aggregate particles and steel bars with different shapes, volumes, and sizes on the bending fracture of concrete. If the cracking of concrete structures is predicted in advance or monitored in real time by DIC and AE techniques, and the concrete structures are maintained and reinforced, all kinds of unnecessary accidents can be prevented.

In summary, in previous studies, DIC and AE technologies were mainly used to study the development of FPZ in the process of concrete fracture damage and to analyze the damage degree of specimens. However, the development law of cracks in concrete in the process of splitting-tensile resistance is still unclear. Moreover, due to the disordered and random distribution of steel fibers, concrete exhibits different microstructure and damage characteristics, so it is necessary to further clarify the crack resistance and strengthening mechanism of steel fibers in the process of splitting-tensile resistance. To provide the relationship between the overall splitting-tensile failure process of SFRC and the local energy dissipation and crack propagation, the splitting-tensile failure process of concrete with four different volume fractions of steel fibers (0.0%, 1.0%, 1.5%, 2.0%) was monitored by combining the AE and DIC techniques in this study. The full-field horizontal displacement and the parameters such as AE energy were obtained, and the influence mechanism of the volume fraction of steel fibers on the splitting and tensile properties of concrete was studied.

## 2. Materials and Methods

### 2.1. Materials

Conch brand P.O 42.5 ordinary Portland cement produced by Liquan Conch Cement Factory in Xianyang, China was used in this test. The physical and chemical properties of the cement are shown in Table 1 and Table 2, respectively.

**Figure 1:**
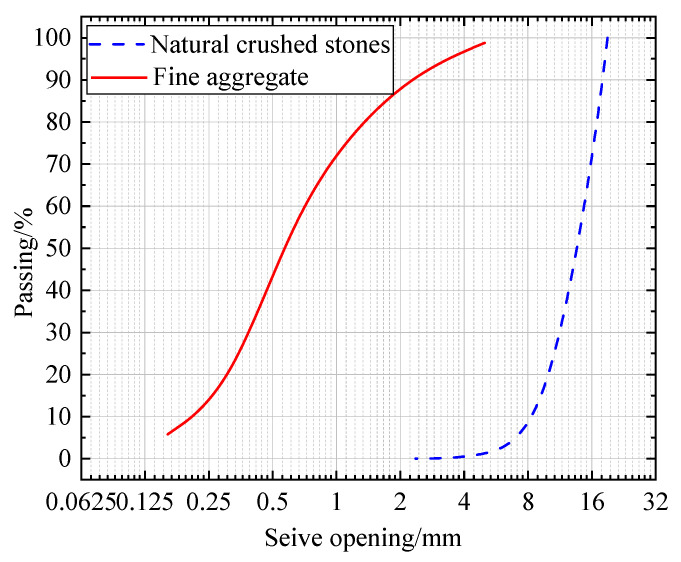
Particle size distribution graph of the aggregate.

The particle size distribution graph of the aggregate as shown in Figure 1. The fineness modulus of the sand used in this time is 2.53, which is the middle sand in zone II. This is according to the “Specification of Sand for Building”, (GB/T14684-2011), which is in line with the fineness modulus range of ordinary concrete sand: *μ_f_* = 3.7~1.6. Coarse aggregate used in this test is 5 mm~20 mm continuously graded gravel, which meet the standard requirements of “Specification of Pebble and Crushed Stone for Building” (GB/T14685-2011).

According to the Chinese standard “Fly Ash Used for Cement and Concrete” (GB 1596-91), fly ash is Grade I, with an average particle size of 12.71 μm and is produced by Shaanxi Weihe Power Plant. Its chemical properties are listed in Table 3.

The water reducer was produced by Shaanxi Qinfen Building Materials Co., Ltd. (Weinan, China). Its physical properties are listed in Table 4. The dosage range of the water reducer is 0.6%~0.8%; in this study, the value is 0.7%. This is in accordance with the “Water Reducing Admixture Used for Concrete-Quality Requirements and Testing Methods” (JGJ 56-84).

The air-entraining agent used was produced by Jiangsu Sobute New Material Co., Ltd. (Nanjing, China), and the content was 0.01%.

**Figure 2:**
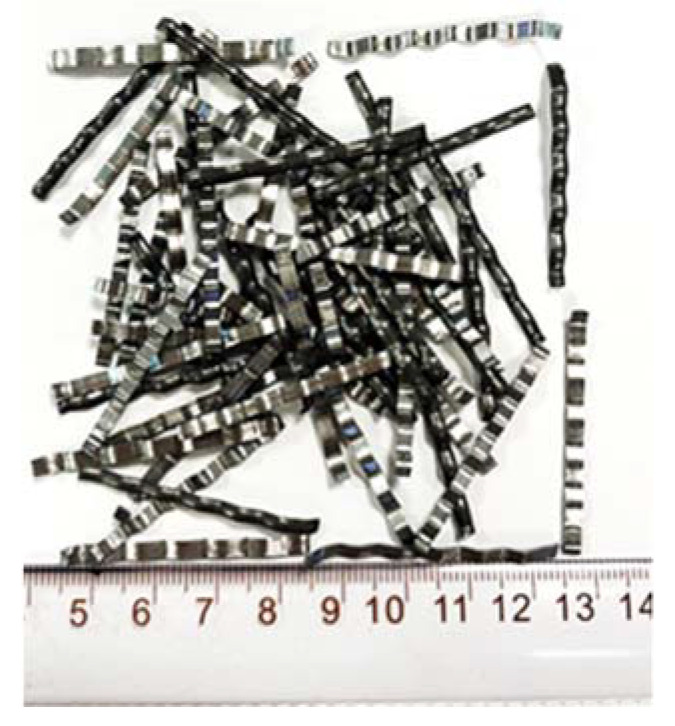
The steel fibers.

The milled steel fiber used was 2.0~2.6 mm in width, 0.4~0.8 mm in thickness, and about 38 mm in length, and was produced by Hengshui Junye Road and Bridge Maintenance Engineering Co., Ltd. (Hengshui, China), as shown in Figure 2.

Four different contents of steel fibers (0.0%, 1.0%, 1.5%, 2.0%) were used in the test. The mix proportions are listed in Table 5.

### 2.2. Workability of Fresh Concrete

According to the “Standard for Test Method of Performance on Ordinary Fresh Concrete” (GB/T 50080-2002), the slump and air content of the fresh concrete were measured. The test results are shown in Table 6. Compared to the concrete without steel fibers, the slump of SFRC decreased as the volume fraction of steel fibers increased. With the increase of steel fiber volume fractions, the air content of concrete decreased firstly and then increased slightly, which could be explained as when the steel fiber volume fraction was less than 1.5%, the addition of steel fibers increased the compactness and decreased the porosity of concrete. However, when the steel fiber volume fraction was more than 1.5%, the steel fibers began to clasp, the workability of concrete decreased, and the porosity increased.

### 2.3. Preparation of Specimens

**Figure 3:**
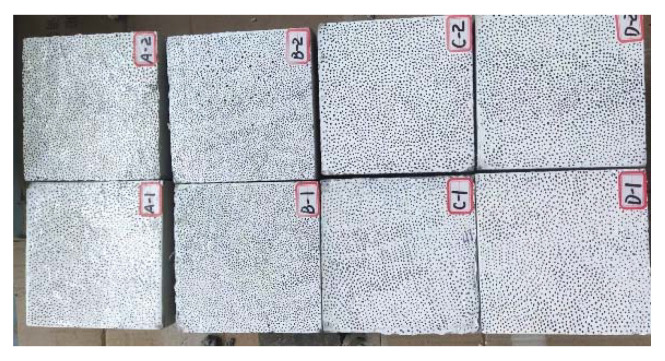
The typical SFRC specimens.

Specimens with dimensions of 150 mm × 150 mm × 150 mm (three specimens for each mix group) were prepared for testing the splitting-tensile strength of SFRC. The SFRC specimens were prepared in the following steps:

(1) Firstly, the premixed mortar with a water-binder ratio of 0.4 was used to paint the HJS-60 double-horizontal axis concrete mixer manufactured by Cangzhou Yixuan Test Instrument Co., Ltd. (Cangzhou, China) to avoid water absorption.
(2) Cement, fly ash, sand, coarse aggregates, and water reducer agent were added into the mixer and mixed at a low speed of 62 rpm for 30 s. After that, steel fibers were dispersed manually into the mixer and blended at a high speed of 125 rpm for 60 s. Finally, the air-entraining agent was dissolved into water and then the liquid was added into the mixer and mixed for 120 s.
(3) The fresh concrete mixture was poured into moulds. The molds were then placed on a vibrating table and vibrated for 30 s to avoid segregation of fresh concrete. The specimens were demolded 24 h after casting and then cured for 28 days in a curing box with a temperature of 20 ± 2 °C and a relative humidity of 95%.
(4) After 28 days’ curing, the specimens were taken out and painted by white-black speckles. The typical specimens prepared are shown in Figure 3. A, B, C and D represent concrete with steel fiber content of 0.0%,1.0%,1.5% and 2.0%, respectively. There are three specimens for each mix group, “A-1” represents the first specimen in the mix group with steel fiber content of 0.0%.

### 2.4. Testing Procedure

**Figure 4:**
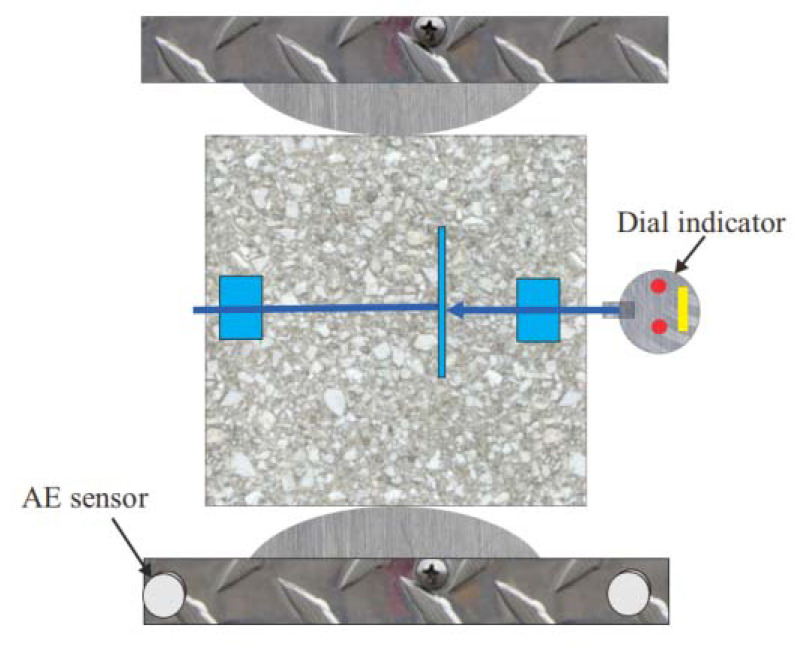
The arrangement diagram of the AE sensor and dial indicator on a concrete specimen.

The splitting-tensile test, with reference to GB/T 50081-2019 [27], was carried out by arranging a dial indicator with a least count of 0.001 mm on the surface of a standard specimen with dimensions of 150 mm × 150 mm × 150 mm to measure its displacement change in the horizontal direction, as shown in Figure 4.

**Figure 5:**
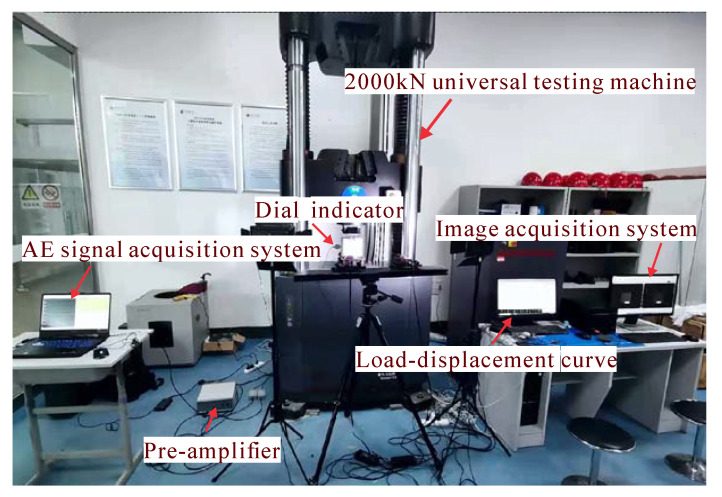
Testing equipment.

The splitting-tensile test was performed on a MTS2000 kN universal testing machine produced by Shanghai Qingbo Test Equipment Co., Ltd. (Shanghai, China) by applying a load at a rate of 0.05 MPa/s and plotting the load-vertical displacement curve by computer, as shown in Figure 5. The AE test instrument was a 2019 version of DS2-type AE monitoring system, which is mainly composed of sensors, preamplifiers, AE acquisition instruments, and a host. The AE sensors were symmetrically arranged on both sides of the bottom plate of the press with a channel threshold of 20 mV. Before the AE test, the AE sensor was bonded with the specimen’s surface by using hot melt adhesive, and the silica-grease coupling agent was coated between the sensor and the specimen to reduce the leakage of the AE signal. A lead fracture test was carried out before each test to verify the coupling effect [28].

The DIC technique was to monitor the splitting-tensile process with the 2018 version of MatchID-2D non-contact, full-field strain measurement and simulation optimization analysis system developed by Match ID company. Two cameras with a resolution of 2048 × 2048 were fixed on a tripod and placed vertically 2 m away from the surface of the specimen being photographed. The crack was expected to occur in the center of the specimen, so the camera’s horizon was focused there [29]. The camera was set to collect five photos per second to record the process of crack development. The equipment was calibrated with a dedicated calibration plate (35 mm) [30]. Finally, a Match ID-stereo analysis system was used for post-processing analysis of the data.

While starting the tester, the DIC system and AE system were triggered at the same time to dynamically track the crack opening and propagation of the specimen during the splitting and tensile process; the loading was terminated when the specimen suffered macroscopic damage, and the DIC system and the AE system were stopped at the same time.

## 3. Results

### 3.1. Splitting-Tensile Strength of SFRC

**Figure 6:**
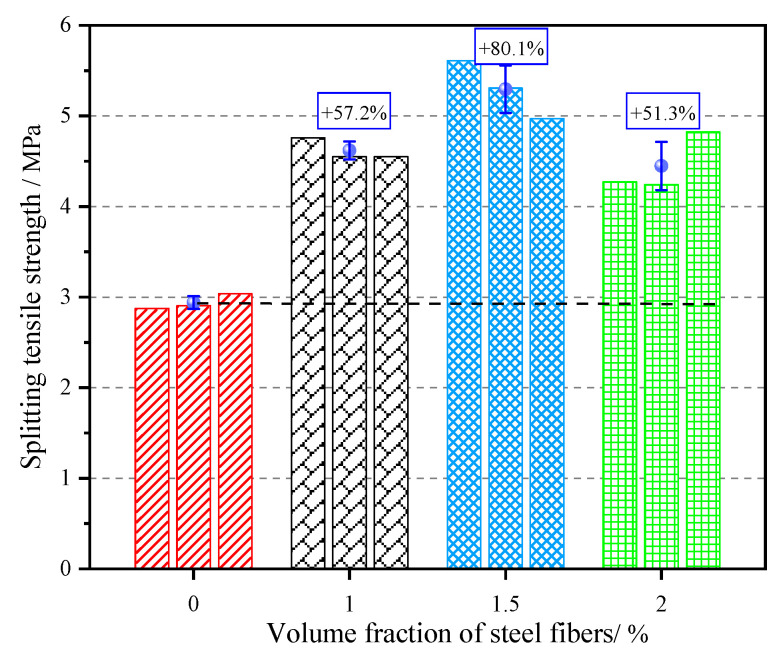
Splitting-tensile strength of SFRC.

The splitting-tensile strength of concrete with four different contents of steel fibers at 28 days of age is shown in Figure 6. According to the figure, the increase in splitting-tensile strength was 57.2%, 80.1%, and 51.3% for the concrete with 1.0%, 1.5%, and 2.0% volume fraction of steel fibers, respectively, compared to that of the reference concrete (0.0% of steel fibers). With the increase in the volume fraction of steel fibers, the splitting-tensile strength of concrete first gradually increased, reaching a maximum value of 5.294 MPa at 1.5% volume fraction of steel fibers, and then decreased significantly. The main reason was that the average spacing between fibers decreased with the increase in the volume fraction of steel fibers, which allowed more fibers to bear the load; at the same time, with the increase in fiber content, the steel fibers were subjected to a certain stress when they were pulled out from the matrix or pulled broken, which delayed the formation and propagation of cracks and improved the splitting-tensile strength of the concrete [31]. When the content of steel fibers exceeded 1.5%, a large number of randomly distributed steel fibers in the concrete hindered the filling of the pores of the coarse aggregate by the cement mortar, leading to an increase in the internal structural voids of the concrete; when the water–cement ratio was constant, the cement paste required to wrap the steel fibers was relatively reduced, resulting in an increase in the internal defects of the concrete [32]. Therefore, the debonding phenomenon occurred in the specimen from the initial stage of loading, and a large number of micro-cracks were generated, which reduced the strengthening effect of steel fibers on the tensile strength of concrete.

### 3.2. Tensile Stress Displacement Relationship during Loading

**Figure 7:**
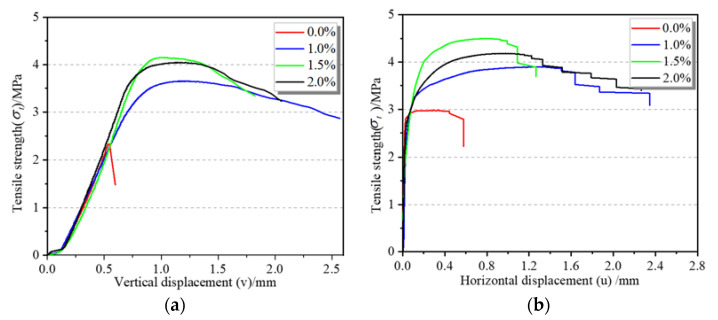
The $\sigma_{t}$-v and $\sigma_{t}$-u curves of SFRC: (**a**) $\sigma_{t}$-v, (**b**) $\sigma_{t}$-u.

The effect of the volume fraction of steel fibers on the $\sigma_{t}$-v curve of concrete is shown in Figure 7a. Comparing the $\sigma_{t}$-v curves of the four types of SFRC, it can be seen that the rising sections of the curves were similar. After the peak stress, the splitting-tensile strength of the reference concrete decreased rapidly, showing obvious brittleness characteristics. With the increase in the volume fraction of steel fibers, the descending section of the curve extended. The $v_{\alpha }$ (vertical displacement corresponding to peak strength) of the reference concrete was 0.53 mm, and then it was increased by 103.9%, 105.8%, and 81.4% when the volume fraction of steel fibers was 1.0%, 1.5%, and 2.0%, respectively. The results demonstrated that the addition of steel fibers mainly affected the curve after the peak strength of the concrete.

The effect of volume fraction of steel fibers on the $\sigma_{t}$-u curve of concrete is shown in Figure 7b. According to the figure, the $\sigma_{t}$-u curves of the concrete with four different volume fractions of steel fibers were similar before the peak strength. After the peak strength, the splitting-tensile strength of the reference concrete suddenly decreased at its horizontal displacement of 0.5 mm, while the strength of the concrete with the volume fraction of steel fibers of 1.0%, 1.5%, and 2.0% decreased slowly with a large increase in horizontal displacement, and the curves showed an obvious stepped shape. The main reason was that after the peak strength, the matrix cracked, and the steel fibers bridging between the cracks bore part of the load; when the fiber axial force increased to the value of the bond failure load, the fibers were pulled out, and then the addition of bridging fibers between the new cracks impeded the sudden drop in the load, and the bridging fibers stopped sliding and pulling. When the strength increased to cause the bridging fibers to slip and pull out once more, the strength suddenly dropped again and the cracks opened, the displacement further increased, and the matrix cracks propagated, with the above process being repeated. However, the initiation strength of the fiber pull-out was lower than that of the previous one due to the decrease in the load-bearing capacity of the fibers that had undergone pull-out and the reduction of the tensile stiffness of the section after crack propagation, so the $\sigma_{t}$-u curve was shown as ladder shaped.

### 3.3. AE Energy Evolution Law in the Test

Griffith [33,34,35] derived the energy balance principle for crack propagation, that is, when crack propagation occurs, the component releases a certain amount of strain energy, part of which is converted into surface energy; for cracks to propagate, resistance needs to be overcome (required surface energy). Crack propagation occurs only when the energy release rate is greater than the resistance. The energy balance theory equation is shown below [36,37,38]:

(1) $$\frac{dW}{dt}=\frac{dU}{dt}+\frac{dT}{dt}+\frac{dD}{dt}$$

where *W* is the work done by an external force on the system, *U* is the initial strain energy, *T* is the strain energy released after cracking, and *D* is the increased surface energy after cracking. When all of *D* was used to form a new crack area *A_t_*, Equation (2) was satisfied:

(2) $$\frac{dD}{dt}=\frac{dD}{dA_{t}}\frac{dA_{t}}{dt}=\gamma_{p}\frac{dA_{t}}{dt}$$

where $\gamma_{p}$ is the surface energy required to form a unit of cracked area.

The energy release rate of crack propagation ($G_{I}$) is computed by using the following equation:

(3) $$G_{I}=-\frac{\pi \delta_{t}{}^{2}a^{2}}{E}$$

where $\sigma_{t}$ is the tensile stress, a is the half length of crack, and *E* is the elastic modulus.

From which the Griffith criterion for crack propagation was derived:

$\frac{d(G-2\gamma_{p})}{da}>0$, instability propagation;

$\frac{d(G-2\gamma_{p})}{da}<0$, crack arrest.

**Figure 8:**
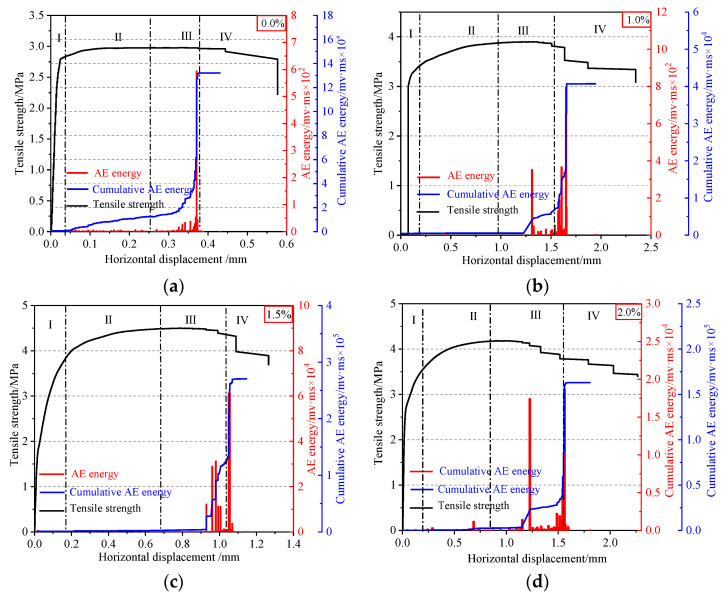
Evolution of AE energy of SFRC specimens: (**a**) 0.0%, (**b**) 1.0%, (**c**) 1.5%, (**d**) 2.0%.

The variation law of AE energy of specimens with horizontal displacement for concrete with four different volume fractions of steel fibers during splitting-tensile process is shown in Figure 8. Based on the tensile stress-horizontal displacement curve, 0.85 $\sigma_{t\max}$, $\sigma_{t\max}$, and 0.9 $\sigma_{t\max}$ after the peak were selected as the critical points to divide the whole splitting-tensile process of SFRC into four stages: (I) At the initial stage of loading, the tensile stress was less than the ultimate tensile stress, and the external input energy was mainly used to compress the internal pores [28,39]. When the internal pores were compacted, the external input energy was stored in the specimen in the form of elastic energy. At this time, there was no crack in the specimen, and almost no AE signal. (II) With the increase in tensile stress, the strain energy released by crack propagation was greater than the required surface energy. Therefore, the crack expanded steadily, and the strain energy released by the crack propagation increased, resulting in a small amount of AE signal. (III) After reaching the ultimate tensile stress, the unstable propagation, interconnection, and penetration of micro-cracks formed a macroscopic main crack surface, with a rapid release of strain energy and fast generation of AE energy. (IV) In stage IV, micro-cracks aggregated and merged to form large-scale cracks. AE signals are no longer generated, indicating that no new cracks are generated and no old cracks are propagated. The energy released by the crack propagation was not enough to meet all the energy required for its propagation, and thus the crack propagation did not continue and the strain energy started to decrease. Compared with the reference concrete, the AE energy accumulation curves of concrete with a steel fiber volume content of 1.0%, 1.5%, and 2.0% were different in stage II and stage III. In stage II, the AE energy accumulation curve of SFRC rose more smoothly; in stage III, the AE energy of SFRC surged more times. The curve increased in steps, and the stepped shape of the curve was more obvious for the concrete with a 1.5% volume fraction of steel fibers. The reason was that the addition of steel fibers reduced the crack generated in the concrete materials at the early stage, and inhibited the crack propagation caused by force failure, which helped the specimen to accumulate more energy before (stage II) failure; at the moment of concrete failure (stage III), most of the steel fibers were pulled apart and pulled out, and the accumulated energy was divided into multiple releases to the outside. The results showed that the reference concrete mostly released a large amount of energy at one time in the splitting-tensile process, while the bridging effect of steel fibers played a moderating role in the splitting-tensile process of concrete, so that the accumulated energy inside the specimen was released in small amounts and multiple times, which improved the brittle properties and the splitting-tensile performance of concrete.

## 4. Discussion

### 4.1. Crack Evolution during Splitting-Tensile Failure of SFRC

**Figure 9:**
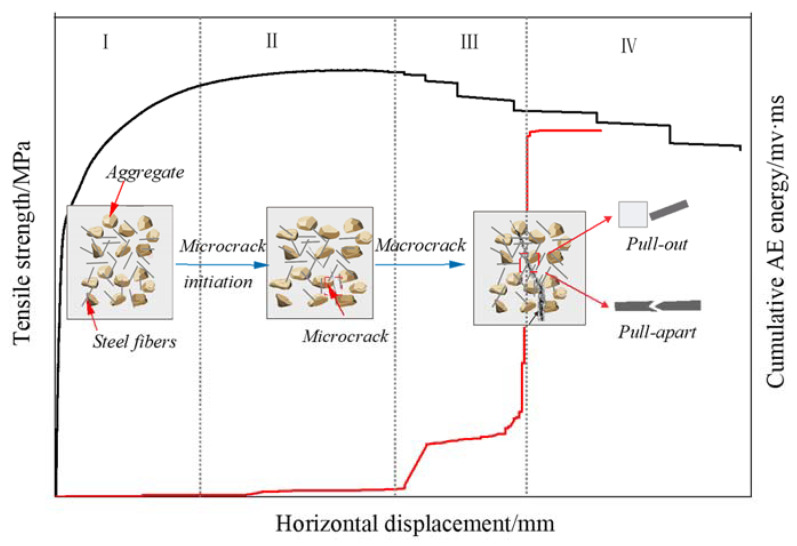
Crack evolution during splitting-tensile failure of SFRC.

The crack evolution process of the concrete with 2.0% volume fraction of steel fibers in the split tensile test is shown in Figure 9. The main failure forms of steel-fiber-reinforced concrete include matrix cracking, fiber/matrix debonding, and fiber breakage [40]. In stage I, with a small load, there was no significant propagation of micro-cracks inside the specimen, the AE activity was less, and the damage of the specimen was light. In stage II, the primary cracks in the concrete propagated and converged, and new cracks were continuously generated, with the main damage type being matrix cracking. In stage III and stage Ⅳ, large-scale cracks spread and permeated rapidly, and finally formed macroscopic cracks inside the specimen, which led to fiber/matrix debonding and fiber fracture. For plain concrete, when the matrix cracked, the crack immediately propagated to the top of the specimen due to the brittleness of the concrete, and the load dropped to zero as the specimen was destroyed. In contrast, with the addition of steel fibers, crack propagation was limited because the material continued to absorb energy and the load did not drop abruptly [41]. Therefore, the addition of steel fibers enhanced the plastic properties of concrete and slowed down the damage process of concrete. Damage to plain concrete was mainly caused by the accumulation of the interface damage between the cement mortar and coarse aggregate and the damage of the cement mortar, while damage to SFRC occurred mainly due to the accumulation of cement mortar damage and fiber tensile fracture damage in its composite system [42].

### 4.2. Pull-Shear Failure Mechanism Based on RA and AF

**Figure 10:**
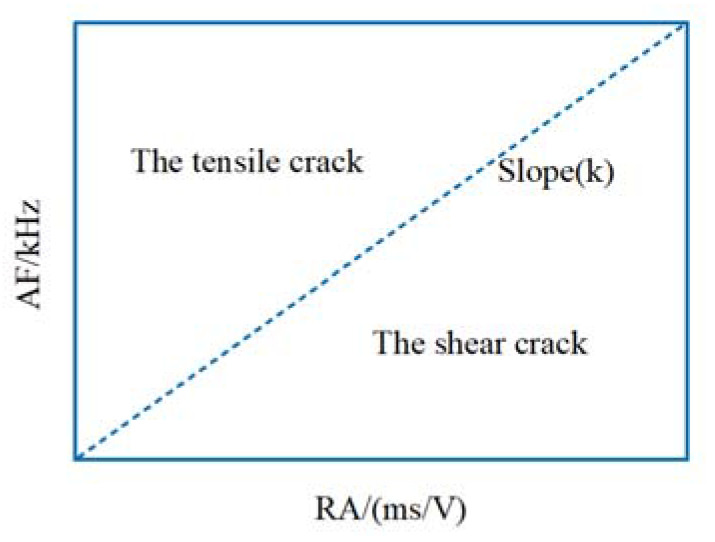
Classification of pull-shear failure based on RA and AF.

Different types of cracks correspond to different AE waveforms. Tensile cracks are generally considered to have low RA values (rise time/amplitude) and high AF values (ring count/duration), while shear cracks have high RA values and low AF values, and tension-shear cracks have low RA values and low AF values [43,44]. The failure mode of the AE signal in concrete can be analyzed according to Figure 10. The discrimination basis of a shear crack and a tensile crack is: RA/AF > 1, shear crack; RA/AF < 1, tensile crack; RA/AF = 1, tensile shear crack.

**Figure 11:**
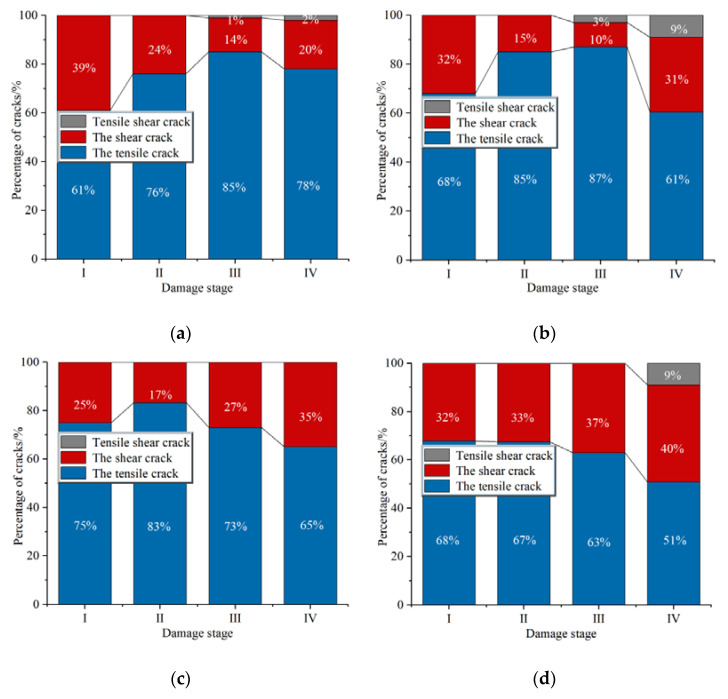
Crack percentage at different stages: (**a**) 0.0%, (**b**) 1.0%, (**c**) 1.5%, (**d**) 2.0%.

The percentage of cracks at different stages of the whole splitting-tensile process for concrete with different volume fractions of steel fibers is shown in Figure 11. As can be seen from Figure 11, tensile failure dominated the splitting-tensile failure process of concrete, and there was a part of shear failure at the same time. The variation law of crack percentage in the splitting-tensile failure process was basically the same for SFRC with 0.0%, 1.0%, and 1.5% volume fractions of steel fibers. In stage I, high shear stress regions appeared at the upper and lower ends of the specimen at the initial stage of loading, resulting in the appearance of a high percentage of shear cracks. From stage II, the percentage of tensile cracks started to grow and remained dominate in the subsequent loading process. Until stage IV, the percentage of shear cracks increased significantly, especially in concrete mixed with steel fibers, which were more than 30%. However, the percentage of cracks in concrete with a volume fraction of steel fibers of 2.0% at each stage was different from that of other concretes, and the percentage of shear cracks increased significantly, ranging from 30% to 40% in each stage. This showed that the excessive content of steel fibers in the concrete easily caused the uneven distribution of steel fibers in the matrix, which reduced the bonding force between steel fibers and the concrete matrix, resulting in shear failure of the concrete in the early stage of loading. This also further explained the reason for the decreased splitting-tensile strength of concrete with a volume fraction of steel fibers of 2.0%.

### 4.3. Variation Law of Full-Field Horizontal Displacement during Splitting-Tensile Process of SFRC

**Figure 12:**
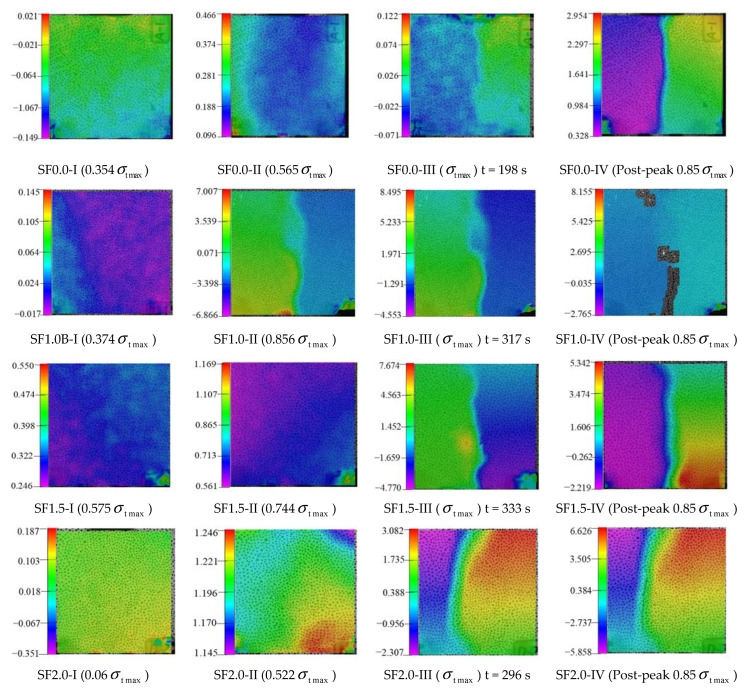
Full-field horizontal displacements of SFRC at different stages of the splitting-tensile test.

The full-field horizontal displacements of SFRC at different stages during the splitting-tensile process are shown in Figure 12. According to the analysis, the cracks developed slowly in the initial stage of loading (stage I), and the micro-cracks first appeared at the restraint and loading of the specimen; there was no significant propagation of micro-cracks due to the small load. With the increase in the load, the cracks gradually extended from the top and bottom of the specimen to the center, and finally connected the specimen (stage II). Therefore, it can be seen that an obvious main crack appeared on the concrete surface at $\sigma_{t\max}$. After the peak load (stage III), the micro-cracks slowly expanded from the vertical centerline to both sides, and the displacement value at the centerline became larger and larger, forming macroscopic cracks located in the centerline region, and the specimen was damaged. The time for the concrete with four different volume fractions of steel fibers to reach the peak load was 198 s, 317 s, 333 s, and 296 s, respectively. With the increase in the volume fraction of steel fibers, the time for concrete to reach the peak load first increased and then decreased, and the longest time was 333 s for the concrete with 1.5% volume fraction of steel fibers, indicating that the addition of steel fiber slowed down the process of concrete damage and failure.

**Figure 13:**
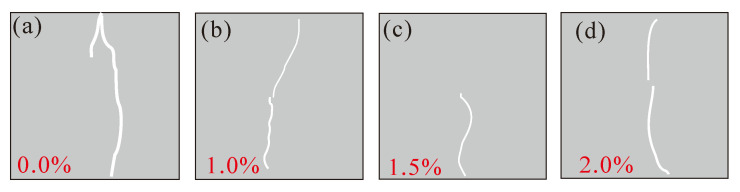
Crack morphology of SFRC after splitting-tensile failure.

Figure 13 shows the crack morphology of SFRC after splitting-tensile failure. The failure form of the concrete specimen after splitting showed that there were cracks on the surface of the reference concrete, and the crack width was relatively large, while the crack size of SFRC was relatively small, and the crack propagation path was tortuous. The formation of cracks in concrete is related to aggregate occlusion. In plain concrete, the strength of cement slurry is lower than the strength of the aggregate, so the cracks will pass through the cement slurry and expand along the edge of the aggregate, resulting in a certain roughness of the crack surface, and aggregate occlusion is produced by shear slip. In SFRC, the crack strike is related to the fiber distribution in the matrix, and the steel fiber stress has a favorable effect on aggregate occlusion, and the concrete specimen was not completely broken when it was damaged; additionally, there was a good bond between the steel fibers and the matrix surface, so the damage interface deflected from the fiber-matrix interface to the matrix surface to produce cracks.

## 5. Conclusions

In this study, the splitting-tensile strength of concrete with four different volume fractions of steel fibers (0.0%, 1.0%, 1.5%, 2.0%) was tested, and the splitting-tensile failure process of concrete was monitored by combining AE and DIC techniques. The evolution law of AE energy and the variation law of full-field displacement of concrete were obtained, and the microscopic failure mechanism of SFRC under macroscopic failure and the crack resistance mechanism of steel fiber were studied. The main conclusions are as follows:

(1) The addition of steel fibers significantly improved the splitting-tensile strength of concrete. With the increase of the volume fraction of steel fibers, the splitting-tensile strength of concrete increased first and then decreased, and reached the maximum value of 5.294 MPa when the content was 1.5%.
(2) AE energy can reflect the four stages of concrete fracture characteristics; the critical state of instability can be judged effectively by the sudden increase of accumulated AE energy. The splitting-tensile process of SFRC mainly included the following four stages: Stage I (0 < $\sigma_{t}$ < 0.85$\sigma_{t\max}$), where there is almost no AE signal; Stage II (0.85 $\sigma_{t\max}$ < $\sigma_{t}$ < $\sigma_{t\max}$), which produces a small amount of AE signal; Stage III ($\sigma_{t\max}$ < $\sigma_{t}$ < post-peak 0.9 $\sigma_{t\max}$), which produces a dense and intense AE energy; Stage IV (post-peak 0.9 $\sigma_{t\max}$ < $\sigma_{t}$), where AE signals are no longer generated. Stage III could be regarded as a precursor to the failure of the specimen.

(3) Tensile cracks dominated the whole process of splitting and tensile failure of SFRC. The percentage of shear cracks in SFRC with volume fractions of steel fibers of 0.0% was high in stage I, 1.0%, and 1.5% was high in stage I and stage IV. However, the proportion of shear cracks in concrete with volume fractions of steel fibers of 2.0% is between 30–40% at every stage.
(4) DIC is possible to measure and accurately determine parameters of crack, such as its actual length, shape, and trajectory of propagation. The combination of AE and DIC is an effective experimental research method, which has great development potential in the field of non-destructive monitoring of SFRC structures.

## Figures and Tables

**Table 1:** The physical properties of the Portland cement.

Cement Type	Stability	Setting Time/min		Compressive Strength/MPa		Flexural Strength/MPa	
		Initial	Final	3 Days	28 Days	3 Days	28 Days
P.O 42.5	Qualified	229	282	28.9	45.0	5.1	7.7

**Table 2:** The chemical properties of the Portland cement.

Material	SiO_2_	Al_2_O_3_	CaO	Fe_2_O_3_	MgO	SO_3_	Ignition Loss
Cement	22.81%	5.62%	61.43%	3.36%	1.35%	2.17%	2.60%

**Table 3:** The chemical properties of the fly ash.

SiO_2_	Al_2_O_3_	CaO	Fe_2_O_3_	MgO	SO_3_	Ignition Loss
49.02%	31.56%	4.88%	6.97%	0.83%	1.2%	4.70%

**Table 4:** The physical properties of the water reducer.

Water Reduction Ratio (%)	Air-Inducing Ratio (%)	Air Volume Fraction (%)	Compressive Strength Ratio (%)			
			1 Day	3 Days	7 Days	28 Days
14	29	5.0~7.0	220	183	180	165

**Table 5:** Mix proportions.

Mix Groups	Composition/kg/m^3^							
	Cement	Fly Ash	Fine Aggregate	Coarse Aggregate	Water	Water Reducer	Air-Entraining Agent	Steel Fibers
0.0%	282	18	544	1240	150	2.1	0.03	0
1.0%	282	18	544	1240	150	2.1	0.03	78
1.5%	282	18	544	1240	150	2.1	0.03	117
2.0%	282	18	544	1240	150	2.1	0.03	156

**Table 6:** The slump and air content of the fresh concrete.

Mix Groups	Slump (mm)	Air Content (%)
0.0%	148	5.2
1.0%	128	2.2
1.5%	116	1.5
2.0%	103	1.6

## Data Availability

The data presented in this study are available on request from the corresponding author.
